# 2389: I-Corps at NCATS: Toward entrepreneurial training for clinical and translational investigators and lessons learned in team-based customer and stakeholder discovery

**DOI:** 10.1017/cts.2017.142

**Published:** 2018-05-10

**Authors:** Molly Wasko, Elaine Morrato, Nicholas Kenyon, Suhrud Rajguru, Bruce Conway, Sara Love, Nate Hafer, Pamela Bhatti, Jonathan Fay, Seth Zonies

## Abstract

OBJECTIVES/SPECIFIC AIMS: The goal of this abstract/presentation is to share lessons learned from participation in the NIH SBIR I-Corps Train-The-Trainer Program, discuss our experiences offering programs at our local institutions, and communicate our plans to develop an I-Corps@NCATS program that can be disseminated across the CTSA network. We believe that an I-Corps@NCATS program will enhance the process of scientific translation by taking best practices from NSF I-Corps and adapting the program to meet the needs of biomedical scientists in academic medical centers. By integrating I-Corps@NCATS training, we hypothesize that the clinical and translational investigator base will be better prepared to identify new innovations and to accelerate translation through commercialization. METHODS/STUDY POPULATION: The diverse, interdisciplinary team of investigators involved in this project span 9 CTSA Hubs, including UAB, Rockefeller, UC Denver, HMC-Penn State, UMass, UC Davis, Emory/Georgia Tech, Miami and Michigan. This team was funded by NCATS in 2015–2016 to participate in the CTSA I-Corps Train-The-Trainer Program in conjunction with the NIH-SBIR/STTR I-Corps national program. The goals were to observe the curriculum, interact with and learn from the NSF National Teaching Team and begin implementation of similar programs at our home institutions. Our I-Corps@NCATS team has been holding monthly, and more recently weekly, conference calls to discuss our experiences implementing local programs and to develop a strategy for expanding CTSA offerings that include innovation and entrepreneurship. Our experience revealed several challenges with the existing NSF/NIH I-Corps program offerings: (1) there is no standard curriculum tailored to academic clinical and translational research and biomedical innovations in the life sciences, and (2) the training process to certify instructors in the I-Corps methodology is a much more rigorous and structured process than just observing an I-Corps program (eg, requires mentored training with a national NSF I-Corps trainer). Our team is proposing to address these gaps by taking best practices from NSF I-Corps and adapting the program to create the I-Corps@NCATS Program, tailored to meet the needs of researchers and clinicians in academic medical centers. RESULTS/ANTICIPATED RESULTS: There are 3 primary anticipated results of our project. First, develop a uniform curriculum for the I-Corps@NCATS Program using the National Teaching Team of experts from the NIH’s SBIR I-Corps program. Second, build the I-Corps@NCATS network capacity through a regional Train-The-Trainer Program. Third, develop a set of common metrics to evaluate the effectiveness and impact of the I-Corps@NCATS Program across the community of CTSA Hubs and their respective collaborative networks. DISCUSSION/SIGNIFICANCE OF IMPACT: Over the past 10 years, CTSA Hubs have accelerated science by creating/supporting programs that provide research infrastructure, informatics, pilot funding, education/training, and research navigator services to investigators. These investments help to ensure that we are “doing science right” using the best practices in clinical research. Even so, it is equally important to make investments to ensure that we are “doing the right science.” Are our investigators tackling research problems that our stakeholders, patients, and communities want and need, to make sure that our investments in science have real-world impact? In order to accelerate discoveries toward better health, scientists need to have a better way to understand the needs, wants and desires of the people for whom their research will serve, and how to overcome key obstacles along the path of innovation and commercialization. To fill this gap, we propose that the CTSA Hubs should include in their portfolio of activities a hands-on, lean startup program tailored after the highly successful NSF Innovation Corps (I-Corps) program. We hypothesize that by adapting the NSF I-Corps program to create an I-Corps@NCATS program tailored to medical research, we will better prepare our scientists and engineers to extend their focus beyond the laboratory and broaden the impact of their research. Investigators trained through I-Corps@NCATS are expected to be able to produce more innovative ideas, take a more informed perspective about how to evaluate the clinical and commercial impact of an idea, and quickly prototype and test new solutions in clinical settings.

